# Identification and validation of a novel cuproptosis-related lncRNA signature for prognosis and immunotherapy of head and neck squamous cell carcinoma

**DOI:** 10.3389/fcell.2022.968590

**Published:** 2022-11-17

**Authors:** Qiu-Shuang Xu, Zheng-Zhong Shen, Ling-Qing Yuan

**Affiliations:** ^1^ National Clinical Research Center for Metabolic Diseases, and Department of Metabolism and Endocrinology, The Second Xiangya Hospital of Central South University, Changsha, China; ^2^ Department of Oral and Maxillofacial Surgery, Center of Stomatology, Xiangya Hospital, Central South University, Changsha, China

**Keywords:** head and neck squamous cell carcinoma, cuproptosis, long non-coding RNAs, prognostic signature, immunotherapy, targeted therapy

## Abstract

Head and neck squamous cell carcinoma (HNSCC) is a highly prevalent and heterogeneous malignancy with a dismal overall survival rate. Nevertheless, the effective biomarkers remain ambiguous and merit further investigation. Cuproptosis is a novel defined pathway of programmed cell death that contributes to the progression of cancers. Meanwhile, long non-coding RNAs (lncRNAs) play a crucial role in the biological process of tumors. Nevertheless, the prognostic value of cuproptosis-related lncRNAs in HNSCC is still obscure. This study aimed to develop a new cuproptosis-related lncRNAs (CRLs) signature to estimate survival and tumor immunity in patients with HNSCC. Herein, 620 cuproptosis-related lncRNAs were identified from The Cancer Genome Atlas database through the co-expression method. To construct a risk model and validate the accuracy of the results, the samples were divided into two cohorts randomly and equally. Subsequently, a prognostic model based on five CRLs was constructed by the Cox regression analysis with the least absolute shrinkage and selection operator (LASSO) algorithm. In addition, the prognostic potential of the five-CRL signature was verified *via* Cox regression, survival analysis, the receiver operating characteristic (ROC) curve, nomogram, and clinicopathologic characteristics correlation analysis. Furthermore, we explored the associations between the signature risk score (RS) and immune landscape, somatic gene mutation, and drug sensitivity. Finally, we gathered six clinical samples and different HNSCC cell lines to validate our bioinformatics results. Overall, the proposed novel five-CRL signature can predict prognosis and assess the efficacy of immunotherapy and targeted therapies to prolong the survival of patients with HNSCC.

## Introduction

Head and neck squamous cell carcinoma (HNSCC) comprises a spectrum of malignancies occurring in various areas, including the oral cavity, the nasopharynx, oropharynx, hypopharynx, and larynx (Haddad and Shin, 2008). In recent years, HNSCC has become a severe threat to human health, and its incidence and mortality rates have been increasing (Sung et al., 2021). In 2020, approximately 930,000 new cases of HNSCC were reported, with 61,900 HNSCC-related deaths globally (Sung et al., 2021). Currently, there is no effective and well-established screening protocol for patients at an early stage. Many patients already reached the middle and advanced stages when they were diagnosed (Machiels et al., 2020). Surgery, radiation, and chemotherapy are currently available therapies for HNSCC. Despite the advancement of numerous therapeutic strategies, the 5-year overall survival of HNSCC is still <50% (Chow, 2020). Hence, it is imperative to explore a stable and valuable prognostic signature to predict the prognosis of HNSCC patients and guide clinical treatment.

As reported in Science, cuproptosis is a novel form of cell death triggered by excessive accumulation of copper. By binding directly to lipoylated components of the tricarboxylic acid (TCA) cycle, copper induces toxic protein stress and, consequently, cell death (Tsvetkov et al., 2022). Copper is a trace element that plays an indispensable role in numerous biological processes. Copper accumulation participates in key features of cancer progression, such as proliferation, metastasis, and angiogenesis (Hanahan and Weinberg, 2011; Denoyer et al., 2015). Over the past decade, growing evidence has proved that dysregulation of copper homeostasis may contribute to the occurrence and progression of multiple cancers, such as prostate cancer, pancreatic cancer, ovarian cancer, breast cancer, and gastric cancer (Blockhuys et al., 2016; Babak and Ahn, 2021; Ge et al., 2022). Previous studies confirmed that patients with oral cancer have significantly higher serum copper levels than healthy controls, and excess amounts of Cu in serum are associated with oral cancer risk (Chen et al., 2019). Furthermore, serum copper levels gradually increased from oral potentially malignant disorder (OPMD) patients to patients with oral cancer, and elevated copper levels were accompanied by marked increased serum ceruloplasmin levels (Jayadeep et al., 1997; Khanna and Karjodkar, 2006). Hosthor et al. (2014) found that the serum levels of copper were correlated with the betel quid chewing habit. Based on the aforementioned findings, cuproptosis may be a significant factor in the development and progression of HNSCC. Nevertheless, the association between cuproptosis and HNSCC is not sufficiently clear. Consequently, the research on cuproptosis has crucial implications, providing us with new avenues for exploring HNSCC.

Long non-coding RNAs (lncRNAs) are widely classified as a subset of untranslated RNAs consisting of more than 200 nucleotides (Batista and Chang, 2013). In recent decades, growing evidence suggests that lncRNAs show a promising potential for regulating cell proliferation, cancer immunity, metastasis, and programmed cell death in tumors (Huarte, 2015; Quinn and Chang, 2016; Zhang et al., 2020; Zhang et al., 2021). Moreover, lncRNAs have been identified as novel potential prognostic indicators for patients with HNSCC (Zhang et al., 2019; Jiang et al., 2022). Nevertheless, at the moment, there are limited studies on cuproptosis-related lncRNAs (CRLs) in HNSCC, and elucidating key CRLs with a prognostic significance in HNSCC patients merits further research.

In this research, we ultimately investigated cuproptosis-associated biomarkers and conducted a CRLs risk model to predict survival outcomes, immune landscape, and chemotherapeutic drug sensitivity in patients with HNSCC. In the future, this signature has the potential to assist doctors in making therapeutic judgments in clinics.

## Materials and Methods

### Data acquisition

We obtained the RNA-seq profiles and clinical features of 339 samples with HNSCC from The Cancer Genome Atlas (TCGA) database (https://portal.gdc.cancer.gov/repository), including 32 normal samples and 325 tumor samples. R (version 4.1.3) was used for the analysis of the entire process. In total, 11 cuproptosis-related genes (CRGs) were acquired from the previous research (Tsvetkov et al., 2022).

### Establishment of the cuproptosis-related lncRNA–mRNA co-expression

Subsequently, the co-expression analysis between CRGs and HNSCC lncRNAs screened 620 cuproptosis-related lncRNAs (|cor| > 0.4 and *p* < 0.001). Then, we screened differentially expressed lncRNAs between tumor and normal samples using the limma package. Differentially expressed lncRNAs satisfied the following criteria: *p* < 0.05 and |log2FC| > 1 (FC, fold change). To examine the degree of correlation between CRLs and their corresponding mRNAs, a Sankey diagram was mapped.

### Construction of the cuproptosis-related prognostic signature

First, a total of 342 HNSCC cases were randomly and equally split into training and testing groups at a 5:5 ratio, and the clinical characteristics of the subjects in both groups were similar. Then, we screened cuproptosis-prognosis–related lncRNAs using univariate Cox regression analysis (*p* < 0.05). Based on the screened lncRNAs, the LASSO regression analysis was used to develop the risk model *via* the “glmnet” R package. We then conducted a multivariate regression analysis and identified five CRLs to establish the signature. Subsequently, we calculated the five CRLs’ corresponding coefficients and constructed a risk score formula for HNSCC. The formula for calculating the risk score of each patient is as follows: Risk score=∑coefi*αi . αi and coefi represented the expression level of each prognostic lncRNA and its corresponding coefficient, respectively.

### Validation of the lncRNA risk model

According to the median value of the risk scores, the samples in each group were subgrouped into high- and low-risk groups. The Kaplan–Meier survival analysis was applied to contrast the overall survival (OS) between the two risk subsets *via* the “survminer” R package. The receiver operating characteristic (ROC) curve was plotted to assess the predictive power of our signature. A principal component analysis (PCA) was performed to analyze the distribution of high- and low-risk groups. To assess the feasibility of the model, the prognostic signature was evaluated within the testing cohort and the entire cohorts. To determine the independence of the CRLs signature, the univariate and multivariate Cox regression analyses were carried out on the risk score and clinicopathologic factors (gender, age, grade, and TNM stage).

### Establishment and assessment of the nomogram

Using the R packages “rms” and “regplot,” we constructed a nomogram based on the five-CRL signature, which incorporated the signature, age, and stage information. Based on the nomogram, we estimated the prognosis of HNSCC patients at 1, 3, and 5 years. The accuracy and reliability of the nomogram were appraised by plotting the calibration curves.

### Functional and pathway enrichment

We identified differentially expressed genes (DEGs) in the high- and low-risk subgroups using the “limma” package (log2|FC| > 1; *p* < 0.05). Gene ontology (GO) and Kyoto Encyclopedia of Genes and Genomes (KEGG) enrichment analyses (Kanehisa et al., 2016) were conducted using the “clusterProfiler” package for functional and pathway enrichment analysis. Additionally, the gene set enrichment analysis (GSEA) (Subramanian et al., 2005) was applied to identify the top five significant pathways in the high-risk and the low-risk subgroups.

### Immunoassay

To explore the association between the risk score and tumor-infiltrating immune cells, we applied seven algorithms, namely, CIBERSORT (Newman et al., 2015), CIBERSORT-ABS (Newman et al., 2015), EPIC (Racle et al., 2017), MCPcounter (Becht et al., 2016), quanTIseq (Finotello et al., 2019), TIMER (Li T.et al., 2020), and xCell (Aran et al., 2017), to estimate the immune infiltrating statuses. Furthermore, the differences in immune cells and immune-related function between the high- and low-risk subsets were studied using the single-sample GSEA (ssGSEA). Lastly, we investigated the expression of the immune checkpoint genes in the two risk subgroups.

### Tumor mutational burden analysis

In addition, the somatic mutation data on HNSCC samples were downloaded from TCGA somatic mutation database. Utilizing the “maftools” package, we analyzed the tumor mutational burden (TMB) of HNSCC samples in the high- and low-risk subgroups. Based on the median level of TMB, patients with HNSCC were subgrouped into high- and low-TMB subsets, and the Kaplan–Meier survival curve was plotted.

### Drug sensitivity analysis

To predict the potential antineoplastic agents that may be applied in HNSCC therapy, the “pRRophetic” R package was utilized to predict the IC_50_ value of common chemotherapy drugs for HNSCC.

### Validation of the quantitative real-time polymerase chain reaction

The paired OSCC (oral squamous cell carcinoma) tumors and tumor-adjacent normal tissue samples were harvested from six OSCC patients in the Oral and Maxillofacial Surgery Department of Xiangya Hospital from September 2022 to October 2022. The freshly obtained tissues were immediately stored at −80°C. Signed informed consent was received from all the OSCC patients, and this study was approved by the Medical Ethics Committee of the Xiangya Hospital of Central South University. Two human HNSCC cell lines (SCC4 and CAL27) and the normal human oral epithelial cell line NOK were purchased from the American Type Culture Collection (ATCC) (Manassas, VA, United States). In brief, we extracted total cellular RNA using the TRIzol reagent (Genstar, Beijing, China). Then, the quantitative real-time PCR (qRT-PCR) experiment was conducted using the SYBR Green Master Mix (Cat # RR047A-5, TaKaRa, Japan), according to the manufacturer’s instructions. The sequences of primers used in our study were as follows:

CDKN2A-DT,forward: 5′-CAG​CGT​GGA​CAG​GAG​CAT​CT-3′,
reverse: 5′-TGT​GAG​GTT​GCG​AAT​GAC​TGC-3′;
AC091982.3, forward: 5′-GGT​TGC​TCT​GCT​CGG​AAG-3′,
reverse: 5′-CCT​GGT​CAC​ATG​CCT​ATG​C-3′;
THAP9-AS1, forward: 5′-CGC​CAT​CGT​CCT​TCT​TGT​GA-3′,
reverse: 5′-GCT​TCC​CCA​TTA​TCT​CCG​CA-3′;
AC010618.2, forward: 5′-GTG​TGA​TGG​CGT​GAT​GGT-3′,
reverse: 5′-GGT​TCA​AGC​GGT​TCT​CCT​G-3′;
GCC2-AS1, forward: 5′-GGC​TCT​GTG​GTA​AGG​CTC​TG-3′
reverse: 5′-ACT​GAC​GAA​TAA​CTT​GGG​GCA-3′;
GAPDH, forward: 5′-GGG​AAG​GTG​AAG​GTC​GGA​GT-3′,
reverse: 5′-GGG​GTC​ATT​GAT​GGC​AAC​A-3′.

### Statistical analysis

SPSS (version 23.0) and R software (version 4.13) were applied for statistical analyses. Wilcoxon rank-sum tests and Student’s t-tests were used to compare the differences between the two subsets. The Kaplan–Meier method was performed to generate survival curves. We visualized the ROC curve and calculated AUC and confidence intervals to assess the model’s accuracy. For each analysis, the statistical significance was set at *p* < 0.05.

## Results

### Identification of cuproptosis-related lncRNAs in head and neck squamous cell carcinoma patients

**FIGURE 1:**
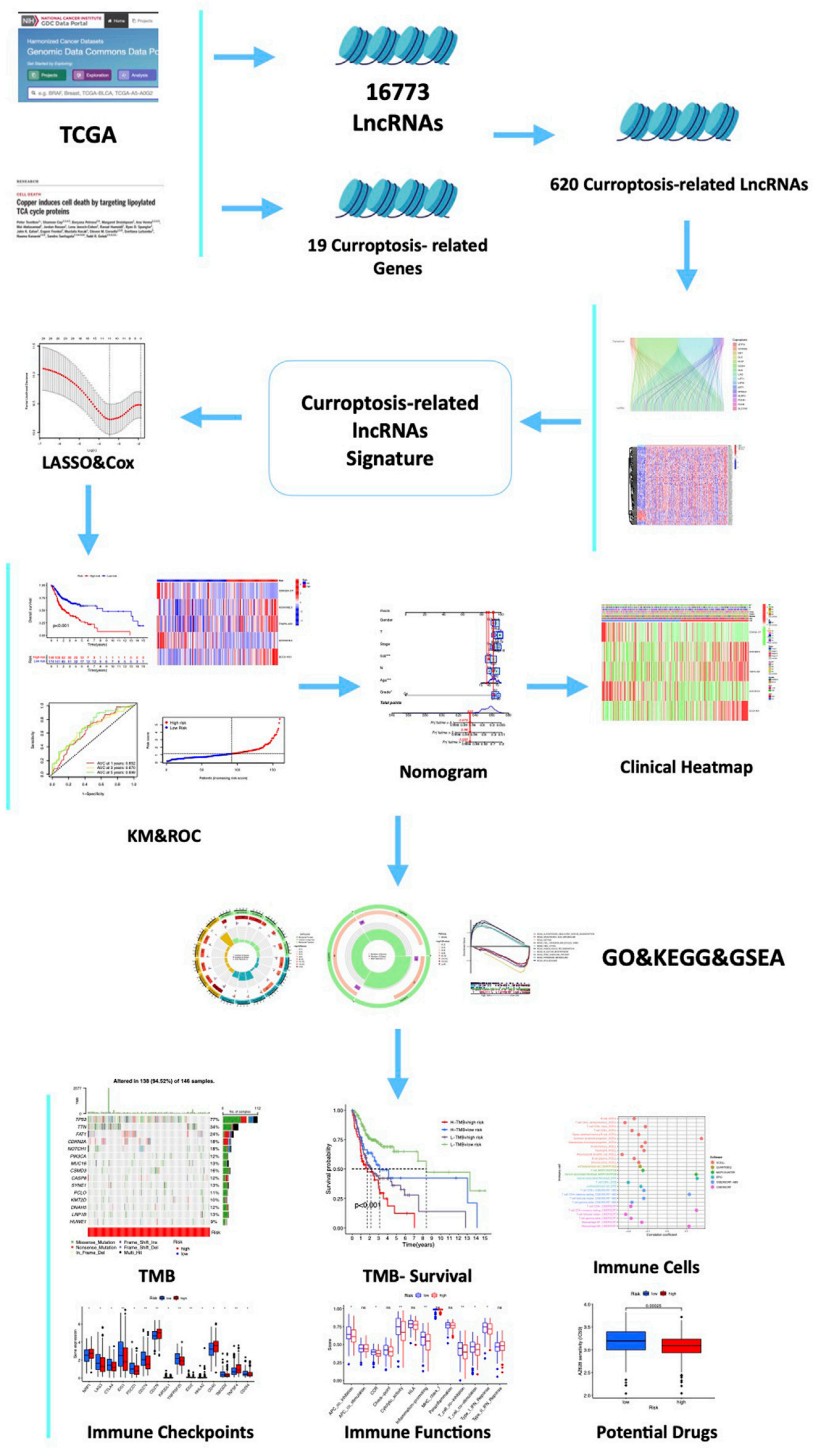
Flow chart of the study. A total of 16,773 lncRNAs and 19 cuproptosis-related genes (CRGs) were acquired from TCGA and previous publications. Then, 620 cuproptosis-related-lncRNAs (CRLs) were identified by the Spearman correlation analysis. Furthermore, a five-CRLs signature was constructed by the Cox regression analysis with the LASSO algorithm. Subsequently, we explored the associations between the signature risk score (RS) and immune landscape, somatic gene mutation, and drug sensitivity. Finally, to explore the expression of these hub CRLs, six pairs of clinical samples and different HNSCC cells were validated.

**FIGURE 2:**
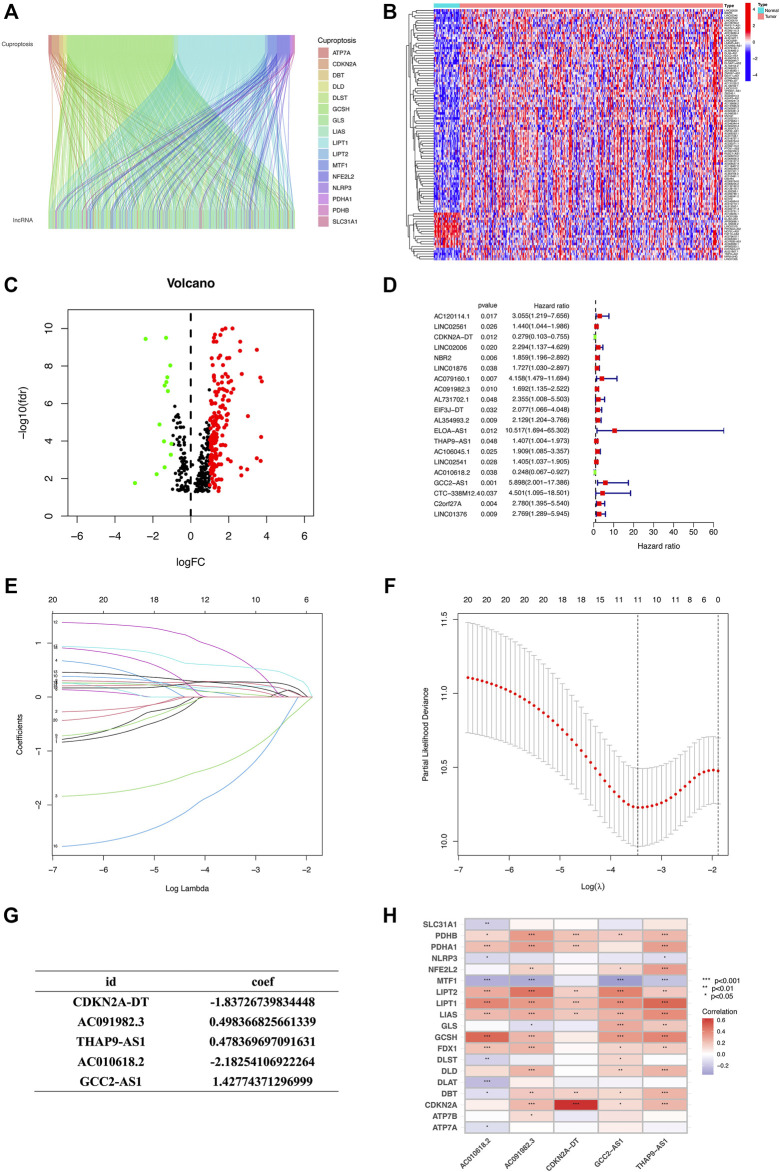
Identification of CR-lncRNAs in HNSCC patients and construction of the CR-lncRNAs prognostic signature. **(A)** Sankey relational diagram for CRGs and CR-lncRNAs. **(B)** Volcano plot of differentially expressed CR-lncRNAs. **(C)** Heatmap for differentially expressed CR-lncRNAs. **(D)** Forest plot of 20 cuproptosis-related lncRNAs identified by the univariate Cox regression analysis. **(E,F)** LASSO regression algorithm identified the risk model. **(G)** Formula of the five-CRL prognostic signature. **(H)** Correlation between five prognostic cuproptosis-related lncRNAs and 19 cuproptosis-related genes in TCGA-HNSCC cohort. The color of each unit showed the degree of correlation. *p < 0.05, **p < 0.01, and ***p < 0.001; ns, no significance.

The flow chart of the research scheme is illustrated in Figure 1. The data for 437 HNSCC samples were collected from TCGA database, which comprised 375 samples of tumor tissues and 32 samples of normal tissues. Additionally, samples with incomplete clinical information were excluded from this study. Following the most recent reported research (Tsvetkov et al., 2022), we identified 19 cuproptosis genes (Supplementary Table S1). The Ensembl gene annotation file identified a total of 19,895 mRNAs and 16,773 lncRNAs. Based on Pearson correlations, 620 lncRNAs were ultimately selected as cuproptosis-related lncRNAs (CRLs). To investigate the potential effects of CRLs on mRNAs, mRNA–lncRNA co-expression network visualization was depicted on the Sankey plot (Figure 2A). Furthermore, 184 differentially expressed lncRNAs (DELs) between normal and tumor tissues were found in TCGA-HNSCC samples, according to the criteria *p* < 0.05 and |log2FC| > 1. Among these DELs, 170 were upregulated and 14 were downregulated (Figures 2B,C).

### Construction and validation of the cuproptosis-related lncRNA prognostic signature

The HNSCC sample data with lncRNA expression data and integral survival information from TCGA database were randomly split into training and testing groups at a 5:5 ratio (Table 1). First, univariate Cox proportional hazard regression analysis was employed to initially screen 20 lncRNAs which were significantly associated with the OS (*p* < 0.05, Figure 2D), including 18 risk lncRNAs, namely, AC120114.1, LINC02561, LINC02006, NBR2, LINC01876, AC079160.1, AC091982.3, AL731702.1, EIF3J-DT, AL354993.2, ELOA-AS1, THAP9-AS1, AC106045.1, LINC02541, GCC2-AS1, CTC-338M12.4, C2orf27A, and LINC01376, and two protective lncRNAs, namely, CDKN2A-DT and AC010618.2. Then, we constructed a LASSO regression model based on the univariate Cox regression analysis using the training set to predict the survival of HNSCC (Figures 2E,F). Five lncRNAs (CDKN2A-DT, AC091982.3, THAP9-AS1, AC010618.2, and GCC2-AS1) were identified for building the optimal prognostic signature. The risk score was calculated as follows: risk score = (−1.8373 × CDKN2A-DT expression) + (0.4984 × AC091982.3 expression) + (0.4784 × THAP9-AS1 expression) + (−2.1825 × AC010618.2 expression) + (1.4277 × GCC2-AS1 expression) (Figure 2G). The heatmap depicted the correlation between 5 CRLs and 19 CRGs, which implied a close relationship between CRLs and CRGs (Figure 2H).

**TABLE 1:** Clinicopathologic characteristics in entire, testing, and training groups.

Covariate	Type	Total	Test	Train	p-value
Age (years)	≤65	203 (62.85%)	104 (65%)	99 (60.74%)	0.4979
	>65	120 (37.15%)	56 (35%)	64 (39.26%)	
Gender	Female	98 (30.34%)	48 (30%)	50 (30.67%)	0.9913
	Male	225 (69.66%)	112 (70%)	113 (69.33%)	
Grade	G1	50 (15.48%)	22 (13.75%)	28 (17.18%)	0.415
	G2	200 (61.92%)	102 (63.75%)	98 (60.12%)	
	G3	62 (19.2%)	32 (20%)	30 (18.4%)	
	G4	2 (0.62%)	0 (0%)	2 (1.23%)	
	Unknown	9 (2.79%)	4 (2.5%)	5 (3.07%)	
Stage	Stage I	20 (6.19%)	8 (5%)	12 (7.36%)	0.1949
	Stage II	55 (17.03%)	28 (17.5%)	27 (16.56%)	
	Stage III	59 (18.27%)	24 (15%)	35 (21.47%)	
	Stage IV	155 (47.99%)	86 (53.75%)	69 (42.33%)	
	Unknown	34 (10.53%)	14 (8.75%)	20 (12.27%)	
T	T0	1 (0.31%)	0 (0%)	1 (0.61%)	0.1911
	T1	32 (9.91%)	12 (7.5%)	20 (12.27%)	
	T2	102 (31.58%)	52 (32.5%)	50 (30.67%)	
	T3	65 (20.12%)	29 (18.12%)	36 (22.09%)	
	T4	97 (30.03%)	56 (35%)	41 (25.15%)	
	Unknown	26 (8.05%)	11 (6.88%)	15 (9.2%)	
M	M0	117 (36.22%)	54 (33.75%)	63 (38.65%)	0.4054
	Unknown	206 (63.78%)	106 (66.25%)	100 (61.35%)	
N	N0	115 (35.6%)	57 (35.62%)	58 (35.58%)	0.1343
	N1	49 (15.17%)	22 (13.75%)	27 (16.56%)	
	N2	103 (31.89%)	59 (36.88%)	44 (26.99%)	
	N3	3 (0.93%)	0 (0%)	3 (1.84%)	
	Unknown	53 (16.41%)	22 (13.75%)	31 (19.02%)	

**FIGURE 3:**
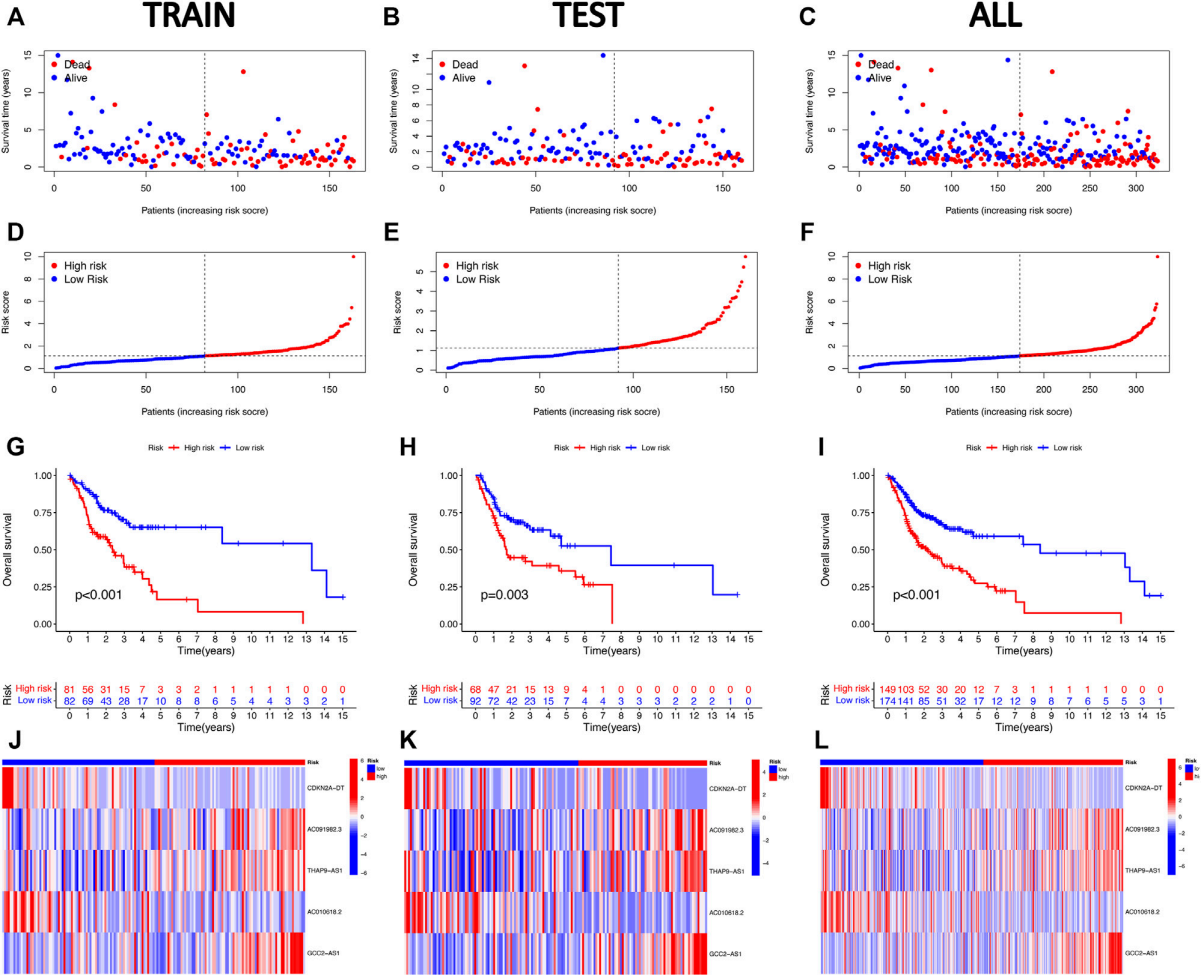
Validation of the five cuproptosis-related lncRNA risk signature for HNSCC. The OS statuses **(A–C)**, risk score distribution **(D–F)**, heat maps of the five CRLs **(G–I)**, and Kaplan–Meier survival analysis for OS **(J–L)** of the high-risk and low-risk groups in the training, testing, and entire subsets.

Accordingly, we calculated the risk score of every HNSCC patient based on the established prognostic model formula, and the patients were categorized into low- and high-risk groups, according to the median value of the risk score. Subsequently, we assessed the prognostic value of this five-CRLs model. The distribution of risk scores, survival time patterns, survival status, and the associated expression of five CRLs was validated in the training, testing, and overall groups (Figures 3A–L). The same trend results were obtained for all three groups of analysis. The scatterplots and risk curves exhibited that the mortality of samples increased significantly with a higher RS. The heat map revealed that three risk lncRNAs were notably upregulated in the high-risk subset, while two protective lncRNAs were markedly downregulated. The K–M curves indicated that the low-risk group had significantly higher survival rates than the high-risk group (training set: *p* < 0.001; testing set: *p* = 0.003; overall set: *p* < 0.001). All of these indicated that the risk prediction model exhibits a good ability to predict.

### An independent head and neck squamous cell carcinoma prognostic indicator of the cuproptosis-related lncRNA signature

**FIGURE 4:**
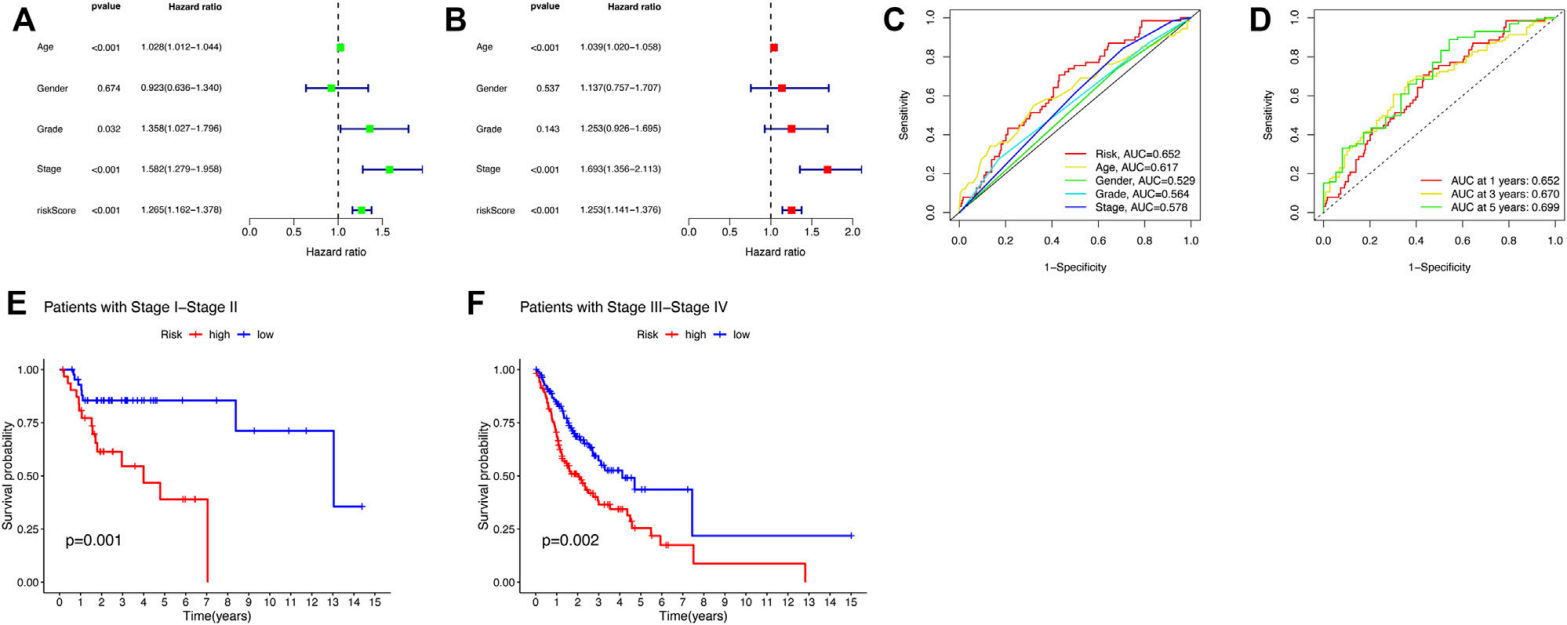
Risk score and nomogram have independent prognostic values. **(A)** Univariate and **(B)** multivariate Cox regression analysis results for HNSCC. **(C)** ROC curve of the prognostic indicators in HNSCC. **(D)** Time-dependent ROC curve of the risk scores in HNSCC. **(E,F)** K–M curves for OC of the HNSCC patients in different clinical stages.

In order to investigate whether the five-CRLs signature is an independent prognostic predictor for patients with HNSCC, a Cox regression analysis was carried out. The univariate Cox regression analysis in the entire group revealed that age, stage, and risk score were directly related to the prognosis of HNSCC (*p* < 0.001) (Figure 4A; Supplementary Table S2). In addition, the multivariate Cox regression analysis further demonstrated that age, stage, and risk score were independent prognostic indicators in HNSCC (*p* < 0.001) (Figure 4B; Supplementary Table S2). The ROC curve was used to assess the predictive ability of the risk signature for the OS of HNSCC patients. The AUC corresponding to the risk score was the highest compared with other clinicopathologic features (Figure 4C). This risk score had a good predictive power at 1, 2, and 3 years (Figure 4D). Above all, these results demonstrated that the risk score based on the five-CRL signature was effective for prognostic evaluation. Moreover, we also examined whether the risk model developed applied to patients at different clinical stages by K–M curves. The results showed that our model was effective not only for early-stage patients but also for late-stage patients (Figures 4E,F).

### Construction and assessment of the prognostic nomogram

**FIGURE 5:**
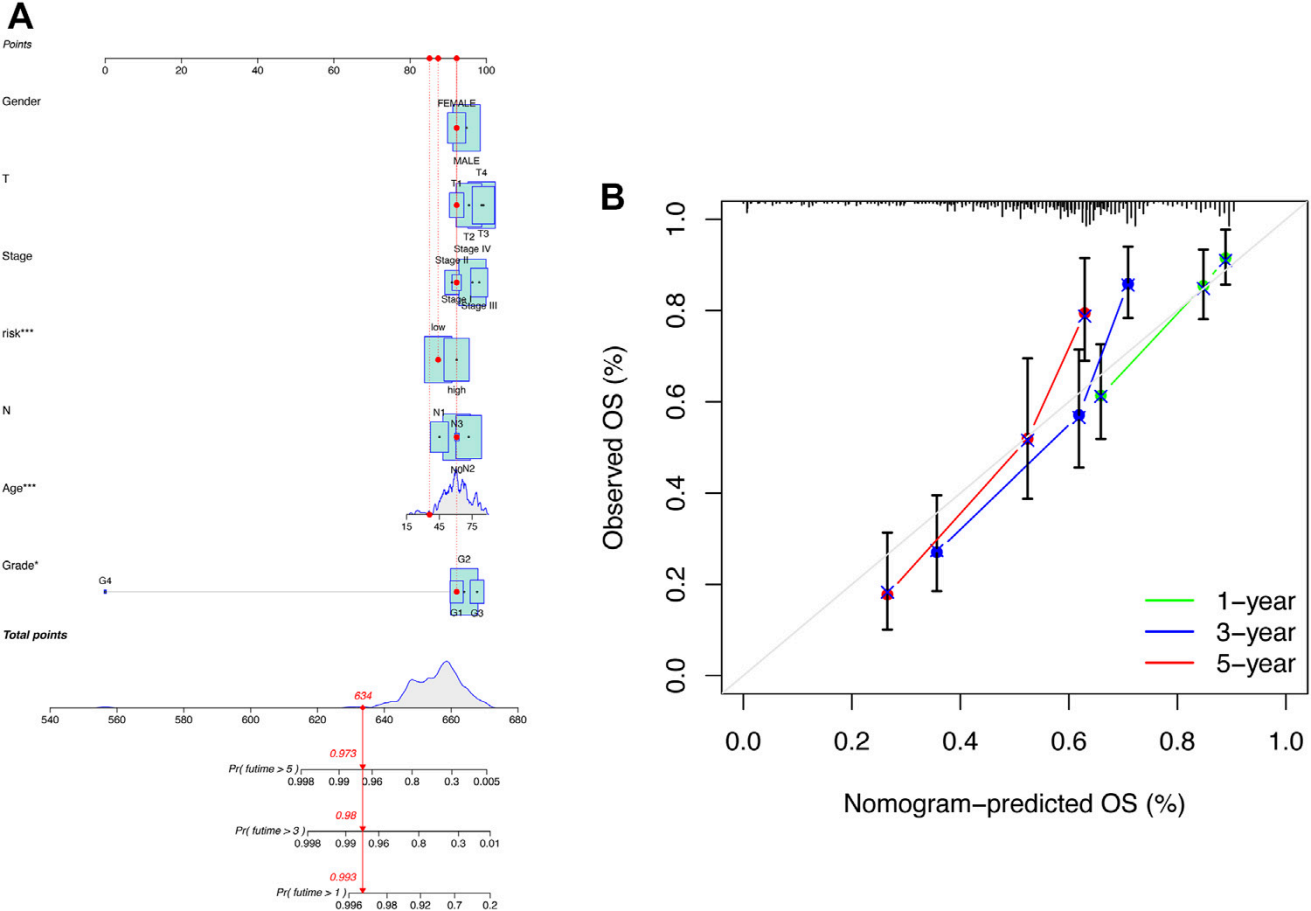
Construction of the nomogram including clinical factors and prognostic cuproptosis-related lncRNAs. **(A)** Nomogram was established for predicting survival outcomes in patients with HNSCC. **(B)** Calibration maps to assess the consistency between the predicted and the actual 1-, 3-, and 5-year OS.

To better apply the signature in clinics, a nomogram comprising the risk score and clinicopathologic information was developed (Figure 5A). The nomogram was fabricated to predict the 1-, 3-, and 5-year OS incidences of patients with HNSCC. Moreover, compared with other clinical features, the risk score of our signature exhibited a better predictive power. The calibration curve revealed that the OS prediction probability predicted by the nomogram was in agreement with the actual observed OS in 1, 3, and 5 years (Figure 5B). These data implied that the nomogram was capable of predicting the prognosis of patients with HNSCC.

### Relationship between the cuproptosis-related lncRNA signature and the clinicopathologic features

**FIGURE 6:**
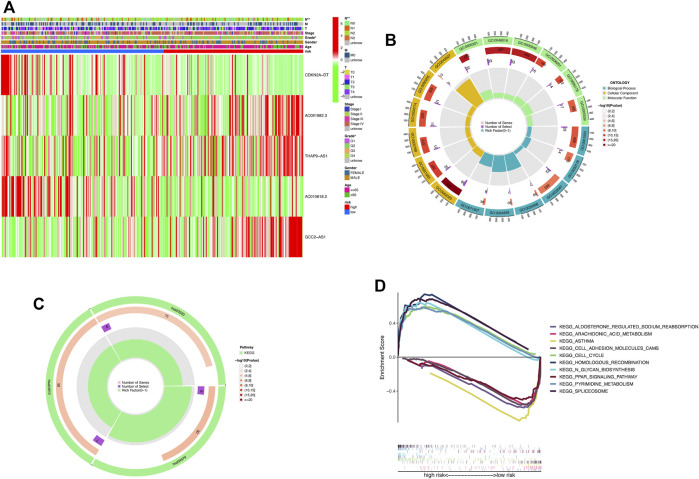
DEGs’ biological functional and pathway enrichment analysis between the two-risk groups based on the prognostic signature. **(A)** Heat map of clinicopathologic manifestations and expression of cuproptosis-related-lncRNAs in the risk groups (green: low expression and red: high expression). **(B)** GO enrichment analysis. BP, biological process; CC, cellular component; and MF, molecular function. **(C)** KEGG pathway analysis. **(D)** GSEA of the notably enriched pathways in the high-risk and low-risk subsets. *p < 0.05, **p < 0.01, and ***p < 0.001; ns, no significance.

We compared the differences in clinicopathologic characteristics between the high-risk and low-risk groups (Figure 6A). The results showed that T-stage, gender, and staging were strongly correlated with risk scores. Taken together, all these results revealed that the model has significant potential in predicting the prognosis of HNSCC patients by assessing their risk scores at the relevant gene expression levels.

### GO, KEGG, and GSEA pathway enrichment analysis

To further explore the differences in biological processes and signaling pathways in DEGs between the high-risk and low-risk subgroups, DEGs with the cut-off criteria of log2|FC| > 1 and FDR <0.05 were selected. The GO analysis revealed that DEGs were involved in ossification, signal release, osteoblast differentiation, and negative regulation of the Wnt signaling pathway at the biological process (BP) category (Figure 6B; Supplementary Table S3). The collagen-containing extracellular matrix, vesicle lumen, endoplasmic reticulum lumen, secretory granule lumen, and cytoplasmic vesicle lumen were enriched in DEGs at the cell component (CC) category. Furthermore, DEGs were mainly associated with the receptor–ligand activity, signaling receptor activator activity, and extracellular matrix structural constituent for the MF category. In the KEGG analysis, these DEGs showed enrichment in cholesterol metabolism, complement and coagulation cascades, and the PPAR signaling pathway (Figure 6C; Supplementary Table S4). Then, we performed GSEA analysis to compare the differences in biological functions and pathways between the high-risk and low-risk subsets (Figure 6D). The results revealed that spliceosome, pyrimidine metabolism, N-glycan biosynthesis, homologous recombination, and the cell cycle were enriched in the high-risk group. Meanwhile, arachidonic acid metabolism, aldosterone-regulated sodium reabsorption, asthma, cell adhesion molecules CAMs, and the PPAR signaling pathway were enriched in the low-risk group.

### Genetic mutation analysis

**FIGURE 7:**
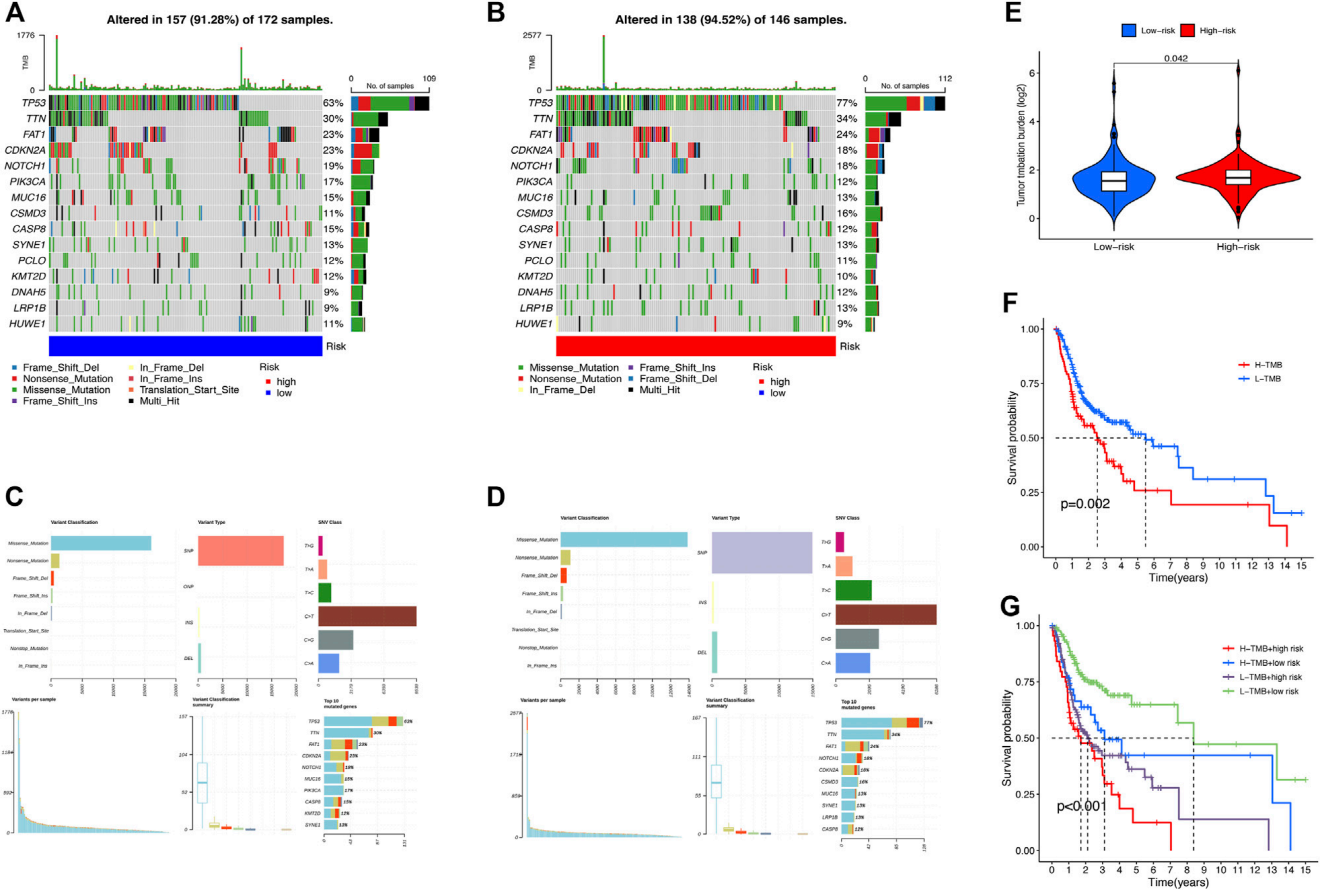
Somatic mutation analysis in HNSCC patients. Waterfall plots **(A,B)** and MAF-summary plots **(C,D)** illustrate the somatic mutation profiles of the high-risk group and low-risk group in HNSCC patients. **(E)** Violin plot of the TMB difference between the high-risk and low-risk patients. **(F)** Kaplan–Meier curve of OS for patients in H-TMB and L-TMB subsets. **(G)** Kaplan–Meier curve of OS for TMB + risk.

First, we examined the differences in patients’ tumor mutational burden (TMB) between the high- and low-risk groups. The top 15 mutated genes of both the groups were shown in a waterfall plot (Figures 7A,B). In contrast to the low-risk subset, more somatic mutations were found in the high-risk subset. The detailed information on somatic mutation is displayed in Figures 7C,D. Additionally, significant differences in TMB existed between the two groups (Figure 7E). According to the median TMB score, samples of HNSCC were divided into high- and low-mutation subsets. The Kaplan–Meier analysis revealed that the patients in the high-mutation group had significantly higher survival rates than the low-mutation group (Figure 7F). Combining the TMB and risk scores to assess the prognosis of HNSCC patients, we noticed that patients with higher TMB in the low-risk subset had the best survival rate, while patients with lower TMB in the high-risk subset had the worst survival rate (Figure 7G).

### Immune landscape of head and neck squamous cell carcinoma patients

**FIGURE 8:**
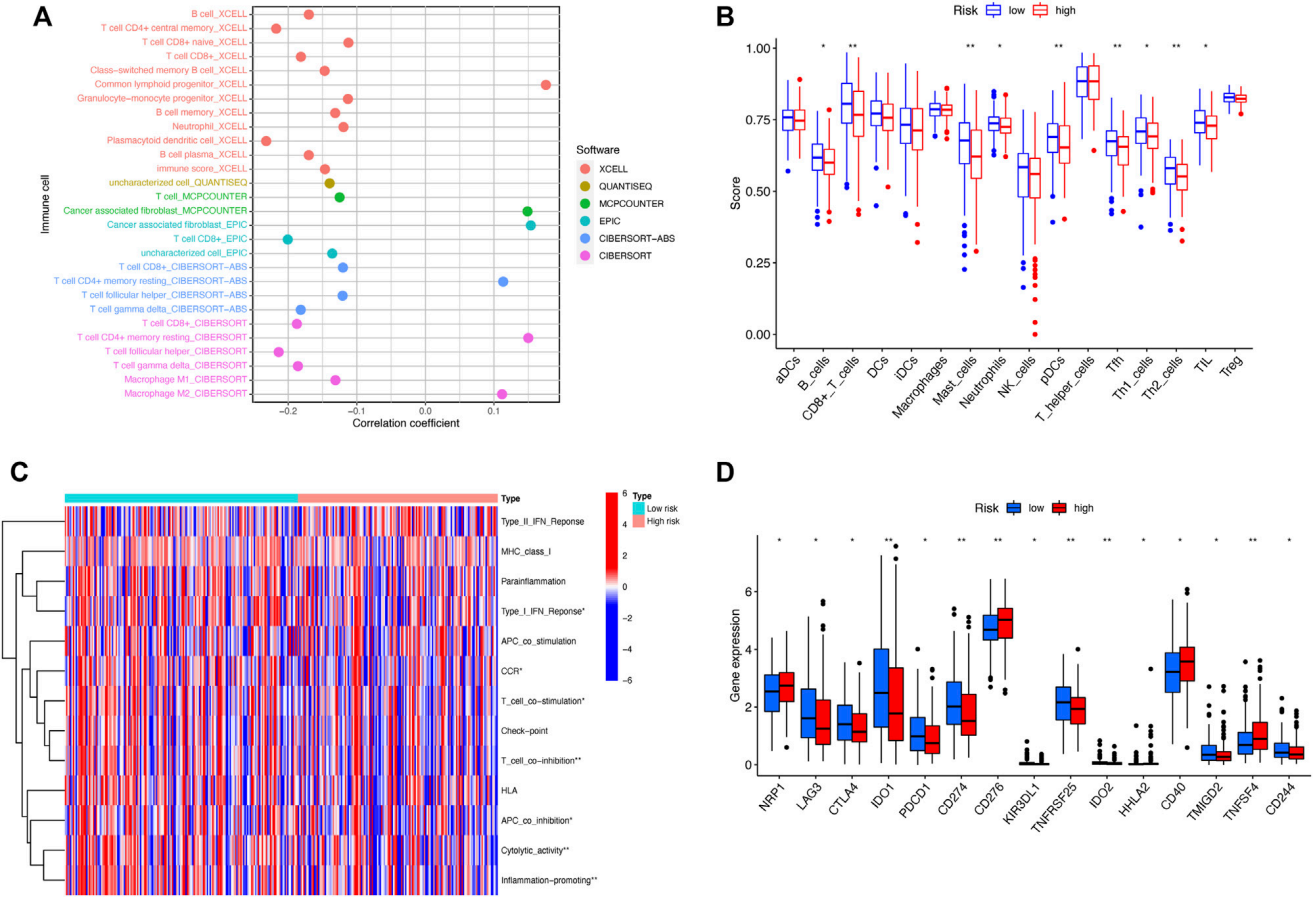
Differences of the immune landscape in two risk subsets of HNSCC. **(A)** Bubble plot depicting the association between the immune infiltration and risk scores *via* CIBERSORT, ESTIMATE, MCPcounter, ssGSEA, and TIMER algorithms. **(B)** Boxplot for the immune cell abundance in high-risk and low-risk subsets. **(C)** Heatmap illustrating the differences of immune-related functions in the two risk groups through the ssGSEA method. **(D)** Boxplot for the comparison of the expression of the commonly immune checkpoints genes among high- and low-risk patients with HNSCC. *p < 0.05, **p < 0.01, and ***p < 0.001; ns, no significance.

The analysis of immune cell infiltration indicated the correlation between risk scores and tumor-infiltrating immune cells. Immune cells were closely associated with high-risk scores on different algorithms (Figure 8A). The ssGSEA of immune cells and functions demonstrated that the patients in the low-risk subset had higher ratios of CD8^+^ T cells, mast cells, plasmacytoid dendritic cells (pDCs), and Tfh and Th2 cells than those in the high-risk subset (Figure 8B). In addition, the examination of immune scores showed that the immune function was enriched in the low-risk group, including the cytolytic activity, T-cell co-inhibition, inflammation promotion, APC co-inhibition, CCR, T-cell co-stimulation, and type I IFN response (Figure 8C). Considering the significance of ICIs for the treatment of tumors, we further explored the immune checkpoint genes in two risk groups. The results showed that the immune checkpoints expressed more activity in the low-risk group, such as IDO1, CD274, TNFRSF25, and IDO2 (Figure 8D). Above all, these findings revealed that low-risk patients might be more sensitive to immunotherapy.

### Sensitivity to clinical drugs between two risk groups

**FIGURE 9:**
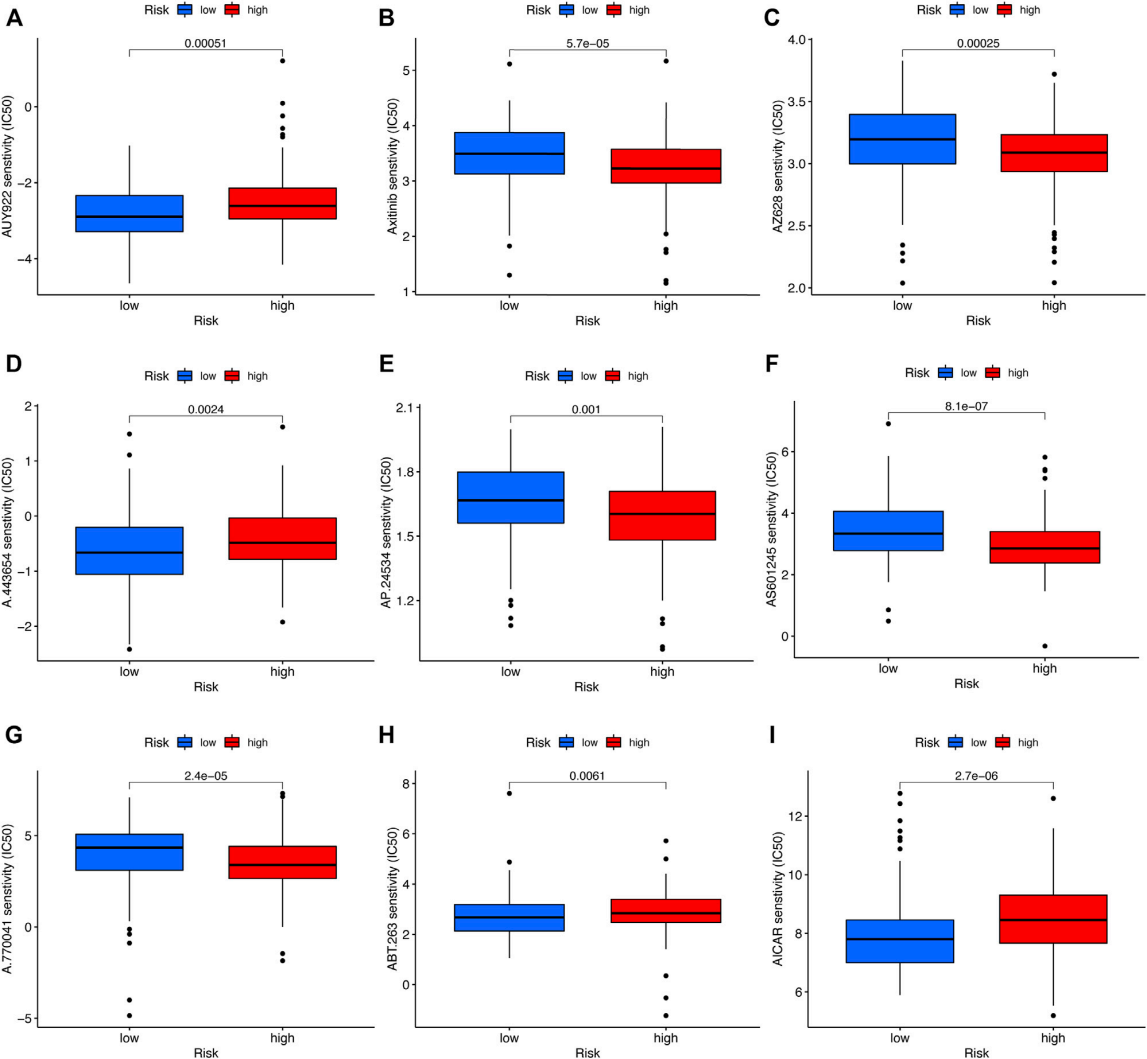
Drug sensitivity analysis. **(A)** AUY-922 (luminespib). **(B)** Axitinib. **(C)** AZ628. **(D)** A.443,654. **(E)** AP.24534 (ponatinib). **(F)** AS601245. **(G)** A.770,041. **(H)** ABT.263 (navitoclax). **(I)** AICAR (acadesine). *p < 0.05, **p < 0.01, and ***p < 0.001; ns, no significance.

To compare the sensitivity of the low- and high-risk groups to conventional targeted drugs, we calculated the IC_50_ values of nine common agents in two subsets (Figures 9A–I). There were statistically significant differences between the two groups (*p* < 0.05). Moreover, these results suggested that A-443654, navitoclax (ABT-263), AICAR (acadesine), and luminespib (AUY-922) may be appropriate for patients with a lower RS based on the five-CRL signature, while AZ 628, axitinib, AS601245, ponatinib (AP24534), and A-770041 may be more suitable for HNSCC patients with a higher RS.

### Validation of cuproptosis-related lncRNA expression

**FIGURE 10:**
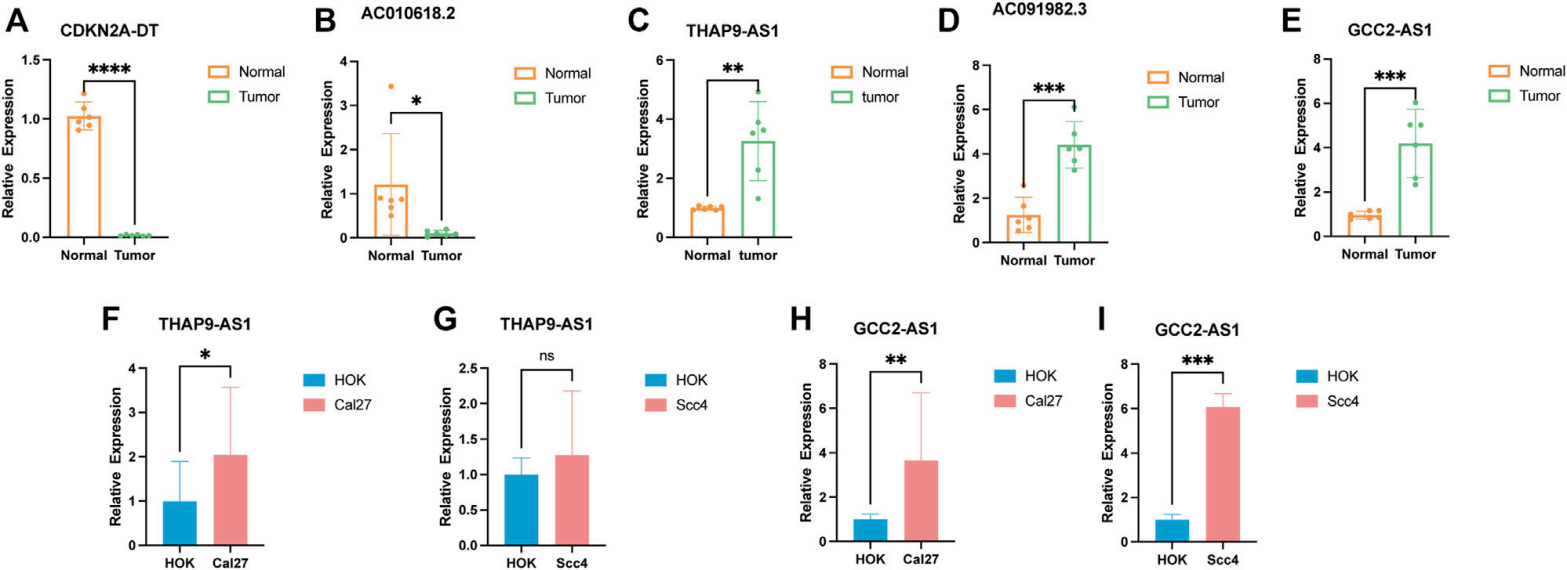
Verification of the expression level of the five cuproptosis-related lncRNAs in tumor tissues and cell lines. **(A–E)** Relative expression of CDKN2A-DT, AC010618.2, THAP9-AS1, AC091982.3, and GCC2-AS1 in six pairs of HNSCC tissues. **(F–I)** Relative expression of THAP9-AS1 and GCC2-AS1 in cell lines. *p < 0.05, **p < 0.01, and ***p < 0.001; ns, no significance.

To verify the findings of the bioinformatics analysis, we conducted RT-PCR on OSCC samples and commonly used cell lines. Our results showed that in six pairs of HNSCC tissues, the expression level of CDKN2A-DT and AC010628.2 was downregulated in tumor tissue samples than in normal tissue samples, and the expression level of THAP9-AS1, GCC2-AS1, and AC91982.3 was upregulated in tumor samples (Figures 10A–E). Moreover, the expression of GCC2-AS1 was significantly increased in SCC4 and Cal27 cells in comparison of the human normal squamous epithelial cell line (NOK) (Figures 10H,I). The expression level of THAP9 was slightly upregulated in Cal27, while there was no difference in THAP9 expression between SCC4 and NOK cells (Figures 10F,G). Furthermore, the expression of CDKN2A-DT, AC091982.3, and AC010618.2 showed no significant differences between tumor and NOK cells. Since TCGA RNA-sequencing data were sequenced on tissues, the results of tissues were more reliable. Overall, the experimental results further validated the reliability of our established risk signature.

## Discussion

In recent years, the prevalence and incidence of HNSCC have increased worldwide, posing a tremendous threat to global human health (Sung et al., 2021). Hence, early diagnosis biomarkers and effective therapeutic strategies are needed for patients with HNSCC (Machiels et al., 2020). Numerous studies have proved that lncRNAs play a significant role in the early diagnosis and pathogenesis of HNSCC (Tang et al., 2013; Zou et al., 2015). Wu et al. (2015) proved that the lncRNA HOTAIR promoted the invasion and metastasis of OSCC by inhibiting E-cadherin. Zhang et al. (2015) found that the high expression of the lncRNA HOTTIP was considered an independent poor prognostic indicator in TSCC. Recently, a novel manner of cell death, cuproptosis, has been reported, which was caused by excessive levels of copper binding to lipoylated elements of the tricarboxylic acid (TCA) cycle, resulting in poisonous protein stress and cell death (Li et al., 2022; Tsvetkov et al., 2022). Researchers found that cuproptosis may play a vital role in the proliferation, metastasis, and angiogenesis of tumors (Denoyer et al., 2015). Nevertheless, the prognostic value of cuproptosis-related lncRNAs in HNSCC remains disputed. This study aimed to develop a novel cuproptosis-related lncRNAs (CRLs) signature to estimate survival and tumor immunity in patients with HNSCC.

In our research, first, we downloaded the transcriptome sequencing data, clinical information, and survival information on HNSCC patients from TCGA. Next, we successfully constructed and validated a novel five-CRLs prognostic model using the Lasso Cox regression. Among these five lncRNAs for signature construction, CDKN2A-DT (CDKN2A-AS1) has been demonstrated to promote epithelial ovarian cancer growth and progression through activating the BMP-SMAD pathway (Zhao et al., 2021). Moreover, CDKN2A-DT may serve as a potential biomarker of unfavorable outcomes in LUAD (Liu et al., 2017). In related research, GCC2-AS1 was found to be significantly overexpressed in lung adenocarcinoma (LUAD), corresponding to promoting the malignant phenotype of cancer cells (Yu et al., 2020). Several studies indicated that the abnormal expression of THAP9-AS1 played a crucial regulatory role in the carcinogenesis of liver cancer, osteosarcoma, esophageal cancer, gastric cancer, and pancreatic cancer (Jia et al., 2019; Li N.et al., 2020; Cheng et al., 2021; Yang et al., 2021; Su et al., 2022). Cheng et al. (2021) reported that the downregulated expression of THAP9-AS1 in esophageal squamous cell carcinoma (ESCC) confers a poor prognosis and suppresses the growth of ESCC xenograft tumors *via* the miR-133b/SOX4 pathway. However, at present, the underlying mechanism of CDKN2A-DT and GCC2-AS1 in HNSCC remains unknown, and there is no documentation for the genes AC091982.3 and AC010618.2. Here, the roles of the five CRLs for our signature establishment in diverse types of tumors are summarized in Supplementary Table S5. In light of our results, these newly identified CRLs may likely contribute to the mechanism of HNSCC and represent promising targets for cancer therapy.

The risk value calculated by the five-CRLs model can be regarded as an independent predictor of the survival of HNSCC patients, just like traditional prognostic indexes such as clinical and pathologic stages. Furthermore, since the risk model is composed of only five identified lncRNAs, it is simpler, easier, and more efficient to apply in the clinical setting to determine patients’ outcomes. Next, we established the nomogram as a tool for predicting the 1-, 3-, and 5-year OS of HNSCC patients. Calibration curves revealed that good consistency was observed between the nomogram predictions and actual outcomes. Statistical analysis demonstrated the high accuracy and sensitivity of our prognostic signature.

Using the median risk score as the threshold, the entire samples were divided into low-risk and high-risk groups based on the risk score. The GSEA analysis revealed that the signaling pathway of pyrimidine metabolism, cell cycle, and glycan biosynthesis ranked high in the high-risk group. In contrast, cell adhesion molecules (CAMs) and PPAR signaling pathways ranked high in the low-risk group. The anti-tumorigenic properties of PPARs are well-known (Harris et al., 2005; Font-Díaz et al., 2021). In gingivo-buccal oral squamous cell carcinoma (GBOSCC), PPARγ is dysregulated and PPARγ ligands can help reduce the occurrence of carcinogen-induced tongue tumors (Das et al., 2019). These findings implied that targeting the PPARγ pathway had potential anti-cancer effects in OSCC. Also, we speculate that the PPARγ pathway may participate in the process of copper-dependent cell death in HNSCC. Nevertheless, these findings should be further investigated.

Numerous research studies have demonstrated that the tumor immune microenvironment plays a crucial role in the formation and progression of HNSCC (Bhat et al., 2021; Qin et al., 2021). In an immunosuppressive tumor microenvironment, HNSCC can escape from immune surveillance through multiple mechanisms (Moy et al., 2017). In our study, the ssGSEA algorithm revealed a significant difference in immune cell infiltration between the high- and low-risk groups. These results demonstrated that cuproptosis was strongly linked with immune infiltration and the tumor-immune microenvironment in patients with HNSCC. In contrast to the high-risk group, the low-risk subset had a higher proportion of immune cell infiltration, such as CD8^+^T-cell and tumor-infiltrating lymphocytes (TILs). These results agreed with previous findings, which showed that CD8^+^ tumor-infiltrating lymphocytes (TILs) contributed to a better prognosis in HPV_DNA+_ p16^INK4a+^ TSCC (Nordfors et al., 2013). Meanwhile, in our research, the patients in the low-risk subgroup exhibited a favorable survival prognosis than those in the high-risk subset. The aforementioned results indicated that high CD8^+^ T-cell infiltration represented good survival outcomes of HNSCC patients.

A revolutionary era of cancer immunotherapy has been inaugurated by immune checkpoint inhibitors, which could positively influence the treatment outcomes of patients with tumors (Mei et al., 2020; Ramos-Casals et al., 2020). Hence, we examined the discrepancies in the expression of common immune checkpoint genes between the high-risk and low-risk groups. The results indicated that the expression of CD276 and TNFSF4 was elevated in high-risk HNSCC patients. Wang et al. (2021) discovered that the immune checkpoint CD276 was highly expressed in cancer stem cells (CSCs), enabling CSCs to evade immune surveillance. TNFRSF4 (CD134/OX40), one of the representative targets of the second-generation immune checkpoints, is considered a promising therapeutic target for antitumor therapy (Bell et al., 2016; Suzuki et al., 2020). Above all, our signature implied that agents blocking these immune checkpoints (CD276 and TNFRSF4) might provide a promising treatment choice for HNSCC patients at a higher risk.

TMB has been confirmed to be a prospective indicator for the effect of immunotherapy (Yarchoan et al., 2017; Eder et al., 2019). Thus, we examined the landscape of TMB in the high-risk and low-risk subsets and found that higher TMB occurred in the high-risk group with a worse prognosis. These findings implied that our signature could filter out the patient candidates for immunotherapy, which would facilitate improving therapeutic outcomes and reducing the risk of immune-related severe adverse events. Furthermore, the two risk groups differed in the sensitivity to common chemotherapeutic agents for HNSCC. These results implied that our model could be valuable in selecting the chemotherapy regimen and evaluating the curative effects.

Undoubtedly, there also existed several limitations and shortcomings in our current research. First, the entire samples on which our signature was performed were derived from TCGA database. Second, the clinical samples for validation of the signature were insufficient. Thus, it is necessary to conduct further research in the following clinical stage. Lastly, the underlying mechanisms of how these CRLs affect cuproptosis in HNSCC should be further explored by *in vivo* and *in vitro* experiments.

## Conclusion

In short, we successfully conducted a novel five-CRL signature to predict survival outcomes in patients with HNSCC, with sound sensitivities and specificities. Moreover, our study provides insights into elucidating the molecular mechanism of CRLs in HNSCC. The signature may help assess the efficacy of targeted agents and immunotherapy and provide guidance for individualized treatment of HNSCC patients.

## Data Availability

The datasets presented in this study can be found in online repositories. The names of the repository/repositories and accession number(s) can be found in the article/Supplementary Material.
