# Supplementary material for: Identification and validation of a novel cuproptosis-related lncRNA signature for prognosis and immunotherapy of head and neck squamous cell carcinoma
Source: Front Cell Dev Biol. 2022 Nov 17;10:968590. doi: 10.3389/fcell.2022.968590 (PMC9712781; doi:10.3389/fcell.2022.968590)
Supplement: Supplementary file 4 [file Table2.DOCX]

**Supplementary Table 2**

**Univariate and multivariate Cox regression analysis in TCGA-HNSCC dataset.**

| Variable | Univariate analysis | | Multivariate analysis | |
| --- | --- | --- | --- | --- |
|  | HR (95% CI) | *p* value | HR(95% CI) | *p* value |
| Age | 1.02774176 | 0.00045128 | 1.038994 | 3.13E-05 |
| Gender | 0.92319601 | 0.67392813 | 1.13655143 | 0.53710594 |
| Grade | 1.35808823 | 0.03193448 | 1.25308783 | 0.14348049 |
| Stage | 1.58229905 | 2.42E-05 | 1.69251479 | 3.38E-06 |
| riskScore | 1.26497529 | 6.61E-08 | 1.25321009 | 2.31E-06 |

HR, hazard ratio; CI, confidence interval
