# Supplementary material for: Identification and validation of a novel cuproptosis-related lncRNA signature for prognosis and immunotherapy of head and neck squamous cell carcinoma
Source: Front Cell Dev Biol. 2022 Nov 17;10:968590. doi: 10.3389/fcell.2022.968590 (PMC9712781; doi:10.3389/fcell.2022.968590)
Supplement: Supplementary file 5 [file Table5.DOCX]

**Supplementary Table 5**

**The functions of the five CRLs in tumors.**

| LncRNAs | Cancer type | Expression | Molecular mechanisms |
| --- | --- | --- | --- |
| CDKN2A-AS1 | Epithelial ovarian cancer (EOC)  (Zhao et al., 2021) | up | CDKN2A-AS1 activated the BMP-SMAD signaling pathway |
| THAP9-AS1 | Esophageal squamous cell carcinoma (Cheng et al., 2021; Pan et al., 2021) | up | 1.THAP9-AS1/miR-133b/SOX4 promoter to activate its transcription.  2.THAP9-AS1 sponging miR-335-5p to regulate SGMS2. |
|  | Osteosarcoma (Yang et al., 2021) | up | THAP9-AS1/miR-133b/SOX4 |
|  | Pancreatic Ductal Adenocarcinoma (Li et al., 2020) | up | THAP9-AS1 plays an important role in PDAC growth by enhancing YAP signaling |
|  | hepatocellular carcinoma (Su et al., 2022) | up | - |
|  | gastric cancer (Jia et al., 2019) | up | - |
| GCC2-AS1 | Lung Adenocarcinoma (Yu et al., 2020) | up | - |

Cheng, J., Ma, H., Yan, M., and Xing, W. (2021). THAP9-AS1/miR-133b/SOX4 positive feedback loop facilitates the progression of esophageal squamous cell carcinoma. *Cell Death Dis* 12(4)**,** 401. doi: 10.1038/s41419-021-03690-z.

Jia, W., Zhang, J., Ma, F., Hao, S., Li, X., Guo, R., et al. (2019). Long noncoding RNA THAP9-AS1 is induced by Helicobacter pylori and promotes cell growth and migration of gastric cancer. *Onco Targets Ther* 12**,** 6653-6663. doi: 10.2147/ott.S201832.

Li, N., Yang, G., Luo, L., Ling, L., Wang, X., Shi, L., et al. (2020). lncRNA THAP9-AS1 Promotes Pancreatic Ductal Adenocarcinoma Growth and Leads to a Poor Clinical Outcome via Sponging miR-484 and Interacting with YAP. *Clin Cancer Res* 26(7)**,** 1736-1748. doi: 10.1158/1078-0432.Ccr-19-0674.

Pan, Q., Li, B., Zhang, J., Du, X., and Gu, D. (2021). LncRNA THAP9-AS1 accelerates cell growth of esophageal squamous cell carcinoma through sponging miR-335-5p to regulate SGMS2. *Pathol Res Pract* 224**,** 153526. doi: 10.1016/j.prp.2021.153526.

Su, Y., Xie, R., and Xu, Q. (2022). LncRNA THAP9-AS1 highly expressed in tissues of hepatocellular carcinoma and accelerates tumor cell proliferation. *Clin Res Hepatol Gastroenterol***,** 102025. doi: 10.1016/j.clinre.2022.102025.

Yang, S., Wang, B., Liu, C., Wang, Q., Wang, R., Su, W., et al. (2021). THAP9-AS1 Promotes Tumorigenesis and Reduces ROS Generation through the JAK2/STAT3 Signaling Pathway by Increasing SOCS3 Promoter Methylation in Osteosarcoma. *Oxid Med Cell Longev* 2021**,** 5620475. doi: 10.1155/2021/5620475.

Yu, F., Liang, M., Wu, W., Huang, Y., Zheng, J., Zheng, B., et al. (2020). Upregulation of Long Non-Coding RNA GCC2-AS1 Facilitates Malignant Phenotypes and Correlated With Unfavorable Prognosis for Lung Adenocarcinoma. *Front Oncol* 10**,** 628608. doi: 10.3389/fonc.2020.628608.

Zhao, Q., Dong, D., Chu, H., Man, L., Huang, X., Yin, L., et al. (2021). lncRNA CDKN2A-AS1 facilitates tumorigenesis and progression of epithelial ovarian cancer via modulating the SOSTDC1-mediated BMP-SMAD signaling pathway. *Cell Cycle* 20(12)**,** 1147-1162. doi: 10.1080/15384101.2021.1924947.
